# Multimorbidity patterns and trajectories in young and middle-aged adults: a large-scale population-based cohort study

**DOI:** 10.3389/fpubh.2024.1349723

**Published:** 2024-05-16

**Authors:** Ignatios Ioakeim-Skoufa, Francisca González-Rubio, Mercedes Aza-Pascual-Salcedo, Clara Laguna-Berna, Beatriz Poblador-Plou, Jorge Vicente-Romero, Helena Coelho, Alejandro Santos-Mejías, Alexandra Prados-Torres, Aida Moreno-Juste, Antonio Gimeno-Miguel

**Affiliations:** ^1^Department of Drug Statistics, Division of Health Data and Digitalisation, Norwegian Institute of Public Health, Oslo, Norway; ^2^Emerging Technologies Advisory Group, ISACA, Chicago, IL, United States; ^3^EpiChron Research Group on Chronic Diseases, Aragon Health Sciences Institute (IACS), Aragon Health Research Institute (IIS Aragón), Miguel Servet University Hospital, Zaragoza, Spain; ^4^Drug Utilisation Work Group, Spanish Society of Family and Community Medicine (semFYC), Barcelona, Spain; ^5^Research Network on Chronicity, Primary Care, and Health Promotion (RICAPPS), Institute of Health Carlos III (ISCIII), Madrid, Spain; ^6^Department of Pharmacology, Physiology, and Legal and Forensic Medicine, Faculty of Medicine, University of Zaragoza, Zaragoza, Spain; ^7^Primary Care Pharmacy Service Zaragoza III, Aragon Health Service (SALUD), Zaragoza, Spain; ^8^Tondela-Viseu Hospital Centre, Viseu, Portugal; ^9^Specialised Section for Regulatory Affairs & Quality, Portuguese Society of Health Care Pharmacists (SPFCS), Coimbra, Portugal; ^10^Aragon Health Service (SALUD), Zaragoza, Spain

**Keywords:** multiple chronic conditions, noncommunicable diseases, multimorbidity patterns, multimorbidity trajectories, multimorbidity evolution, multimorbidity development, metabolic syndrome, systemic chronic inflammation

## Abstract

**Introduction:**

The presence of multiple chronic conditions, also referred to as multimorbidity, is a common finding in adults. Epidemiologic research can help identify groups of individuals with similar clinical profiles who could benefit from similar interventions. Many cross-sectional studies have revealed the existence of different multimorbidity patterns. Most of these studies were focused on the older population. However, multimorbidity patterns begin to form at a young age and can evolve over time following distinct multimorbidity trajectories with different impact on health. In this study, we aimed to identify multimorbidity patterns and trajectories in adults 18–65 years old.

**Methods:**

We conducted a retrospective longitudinal epidemiologic study in the EpiChron Cohort, which includes all inhabitants of Aragón (Spain) registered as users of the Spanish National Health System, linking, at the patient level, information from electronic health records from both primary and specialised care. We included all 293,923 patients 18–65 years old with multimorbidity in 2011. We used cluster analysis at baseline (2011) and in 2015 and 2019 to identify multimorbidity patterns at four and eight years of follow-up, and we then created alluvial plots to visualise multimorbidity trajectories. We performed age- and sex-adjusted logistic regression analysis to study the association of each pattern with four- and eight-year mortality.

**Results:**

We identified three multimorbidity patterns at baseline, named *dyslipidaemia & endocrine-metabolic*, *hypertension & obesity*, and *unspecific*. The *hypertension & obesity* pattern, found in one out of every four patients was associated with a higher likelihood of four- and eight-year mortality (age- and sex-adjusted odds ratio 1.11 and 1.16, respectively) compared to the *unspecific* pattern. Baseline patterns evolved into different patterns during the follow-up.

**Discussion:**

Well-known preventable cardiovascular risk factors were key elements in most patterns, highlighting the role of hypertension and obesity as risk factors for higher mortality. Two out of every three patients had a cardiovascular profile with chronic conditions like diabetes and obesity that are linked to low-grade systemic chronic inflammation. More studies are encouraged to better characterise the relatively large portion of the population with an unspecific disease pattern and to help design and implement effective and comprehensive strategies towards healthier ageing.

## 1 Introduction

Many people today live with two or more chronic diseases. This situation, commonly known by the term multimorbidity (1), is becoming a common clinical presentation of chronicity (2–6); in older adults, it is already the rule (7–12). Multimorbidity challenges patients, clinicians, and healthcare systems worldwide not only because its prevalence is on the rise but primarily due to its tremendous impact on health. People with multiple chronic conditions are susceptible to fragmented care and a higher risk of inappropriate clinical management with, consequently, negative outcomes and adverse events (13–20). Healthcare professionals very often face the need to stretch their competencies to offer an optimal person-centred approach and meet patient’s expectations (21). Decision-makers are pressured to design and implement effective policies aiming to improve population health and minimise unnecessary utilisation of healthcare services and costs. For these reasons—and many others as well—there is an urgent need to advance research in multimorbidity in many different populations and clinical settings (22, 23).

We already know chronic diseases do not occur randomly, but they tend to cluster, revealing the existence of different multimorbidity patterns. The characterisation of these patterns allows us to identify individuals who may benefit from similar clinical interventions that consider multimorbidity to offer optimal clinical management and to improve patients’ safety (21–29). The in-depth study of these patterns may also reveal systematic associations between chronic diseases and give us essential information to generate hypotheses and uncover common underlying aetiopathogenic mechanisms. Recent studies reported various multimorbidity patterns; however, there is a wide variety of methodological approaches to identifying and characterizing them, resulting in discrepancies regarding their nature and prevalence (30–35). Nonetheless, consistent findings show that specific chronic diseases are key in clustering the population based on the individuals’ clinical profile. We find cardiometabolic diseases, mental health problems, and allergies amongst these conditions (30).

So far, most studies have followed a cross-sectional approach (5, 24, 30–42). However, it is crucial to consider the dynamic nature of the clinical profile of an individual. Multimorbidity patterns are not static and may change over time due to many factors. This is of high importance, especially in patients with multiple chronic conditions where, from one side, the systematic associations of chronic diseases reveal potential disease-disease interactions and, on the other side, the high prevalence of polypharmacy may result in a higher risk of drug-disease and drug–drug interactions (43, 44). As the prevalence of multimorbidity in people 60 years of age and older is extremely high, ranging between 55–98% (12), the few studies on multimorbidity trajectories have focused primarily on the older population (45–52). However, multimorbidity is present in all age groups (24). Characterising the evolution of the different multimorbidity patterns in the young and middle-aged population may allow us to address this challenging public health problem earlier, prevent numerous adverse outcomes, and achieve healthy ageing. This large-scale epidemiologic study aims to identify multimorbidity patterns in young and middle-aged adults (between 18 and 65 years) from a population-based cohort and study the evolution of these patterns over time through a four- and eight-year follow-up.

## 2 Materials and methods

### 2.1 Ethical considerations

The study was approved by the Clinical Research Ethics Committee of Aragón (CEICA; protocol number PI17/0024). CEICA waived the requirement to obtain informed consent from the participants due to the epidemiological nature of the project and the use of anonymised data that were presented at an aggregated level.

### 2.2 Study design and setting

We conducted a retrospective longitudinal study in the EpiChron Cohort; the full cohort’s profile has been published elsewhere (53). The EpiChron Cohort includes all inhabitants of Aragón (Spain) registered as users of the Spanish National Health System (as of 1 January 2011, 1,253,292 individuals, 98% of the total inhabitants in the region), linking, at the patient level, information from electronic health records (EHR) from both primary and specialised care, and clinical-administrative databases. All the information is anonymised, and it includes data from primary care, hospital care, specialist care, emergency rooms, pharmaceutical billing, and the user database.

In this study, we included all chronic patients 18–65 years old who were included in the EpiChron cohort and had multimorbidity (≥2 chronic conditions) in 2011. We carried out an eight-year follow-up, from 2011 to 2019, to study the evolution of their clinical profile and identify the different multimorbidity trajectories. The particular cohort for this specific study was a closed cohort; no individuals were added during the follow-up.

### 2.3 Variables

For each patient, we analysed age at baseline, biological sex at birth, all the diseases recorded in the patient’s EHR, medication use, and mortality. Mortality was assessed in 2015 (four-year mortality since baseline) and 2019 (four-year mortality since 2015, eight-year mortality since baseline). Lost to follow-up (inactive status in the user database) was treated as missing data and was excluded from the corresponding cluster analysis in 2015 and/or 2019.

Primary care diagnoses were initially registered according to the International Classification of Primary Care, first edition (ICPC–1) (54), and then mapped to the International Classification of Diseases, ninth edition, clinical modification (ICD-9-CM) (55). We sorted all diagnoses into 226 clinically relevant categories using the Clinical Classifications Software (CCS) (56), which were classified into chronic and nonchronic conditions using the Chronic Condition Indicator software tool, resulting in 153 chronic conditions (57). Chronic conditions were defined as those conditions that last 12 months or longer and place limitations on self-care, independent living, and social interactions, and/or result in the need for ongoing intervention with medical products, services, and special equipment (57). Some of the diagnostic labels were renamed or grouped together to facilitate their clinical interpretation, resulting in a final list of 130 chronic diseases. Multimorbidity was defined as the presence of two or more chronic diseases from this list. For the baseline clinical profile of the participants, we included chronic diseases with active status in the patient’s clinical history in 2011. During the follow-up, we included additional comorbidity in 2015 and 2019.

We studied medication use based on drug dispensations using the World Health Organization’s Anatomical Therapeutic Chemical (ATC) classification system at the fourth ATC level (mainly corresponding to chemical subgroup) (58, 59). Repeated dispensations of drugs that were classified in the same fourth ATC level during a follow-up period were counted once.

To study the potential role of acute conditions and drugs in the evolution of multimorbidity patterns, we included such information during the 2012–2015 and 2016–2019 follow-up periods. As acute diseases, we defined all pathological conditions that were classified as nonchronic in the previous step. The epidemiology, interactions, and role of acute diseases in the population with multiple chronic conditions are poorly studied and thus, in this study, we aimed to identify all the different acute clinical entities that emerged during the follow-up, and to study any possible associations between multimorbidity patterns and acute diseases. Consequently, to estimate the number of acute diseases, diagnoses of acute conditions that were diagnosed two or more times in a follow-up period were counted once.

### 2.4 Statistical analysis

We first conducted cluster analyses in each cross-section (baseline in 2011, and then, in 2015 and 2019) to identify clusters of patients based on the presence/absence of each chronic disease (dichotomous variables), considering all chronic diseases with a prevalence ≥1.0% at the corresponding year. The prevalence of each disease was calculated by dividing the number of patients with a diagnosis registered in their EHR by the total number of patients.

As described in previous studies (38, 60), we first went through an optimisation process to properly select the clustering method. We ran an agglomerative hierarchical method, but because of computational limitations, we finally conducted a *k*-means non-hierarchical analysis. We used the Jaccard index as a measure of similarity, to establish the distance between patients. The number of multimorbidity patterns, their clinical particularities, and their clinical interpretation followed a process in which the clinical partners (II-S, FG-R, AP-T, and AM-J) discussed it in consecutive rounds until a consensus was reached. Regarding the number of multimorbidity patterns in each cross-section, we selected the lowest possible number of patterns that maximised both intra-group homogeneity and inter-group heterogeneity, while offering clinical consistency, and considering the Caliński and Harabasz index (61). To analyse the clinical particularities of each pattern, we first calculated the prevalence ratio (PR) of every single chronic condition in each multimorbidity pattern, comparing the observed prevalence (OP; its prevalence in the pattern) with its expected prevalence (EP; its prevalence in the population) (PR = OP/EP). In general, a chronic condition was considered an essential part of a pattern’s particular clinical profile if: (i) PR ≥2.0 and OP ≥10%, or (ii) OP ≥20% and PR ≥1.5. As all multimorbidity patterns should have at least one chronic disease as essential part of their particular clinical profile, this requirement was also taken into consideration when defining the total number of patterns in each cross-section. When possible, multimorbidity patterns were given a clinical name to improve readability. For consistency, naming was mainly in accordance with the clinical particularities of each pattern. What characterises the clinical particularity of each pattern is the considerably higher observed prevalence of some common diseases in comparison with the expected one; however, these chronic diseases are not necessarily the most prevalent ones in the pattern. In general, all chronic diseases may be present—although at a very low prevalence—in all patterns, or in most of them. Detailed information regarding the clinical composition of all multimorbidity patterns is given in the Supplementary material to enhance clarity and allow for appropriate conclusions and plausible comparisons.

We performed a descriptive analysis of the demographic, clinical, and drug utilisation characteristics of all multimorbidity patterns in each cross-section (2011, 2015, and 2019) and follow-up period (four-year follow-up periods 2012–2015 and 2016–2019, and eight-year follow-up period 2012–2019). For comparisons between patterns, we used Pearson’s chi-squared test for categorical variables, and Student’s t-test and ANOVA test with the Bonferroni multiple-comparison correction to compare means.

We performed age- and sex-adjusted logistic regression analysis to study associations between multimorbidity patterns and mortality. Reference group was selected to be the multimorbidity pattern with the lowest mortality rate. We used age- and sex-adjusted multinomial logistic regression to study associations between the 2015 and 2019 multimorbidity patterns and the diagnoses of acute diseases during the previous four-year follow-up period, using as reference group the pattern with the lowest prevalence in the corresponding year.

All the analyses were conducted in STATA software (STATA, Version 12.0, StataCorp LLC) (62), with the statistical significance set at *p* < 0.05. For visualisation, we used R programming language (R Core Team, 2020), and RStudio (RStudio Team, 2020) to create alluvial plots (63, 64).

## 3 Results

### 3.1 Baseline characteristics of the study population

In 2011, 293,923 individuals 18–65 years old in the EpiChron Cohort had multimorbidity (see Figure A1). Their mean age was 47.77 ± 12.15 (standard deviation) (48.73 ± 11.70 in men; 47.06 ± 12.42 in women) (Table 1). The most common chronic diseases were disorders of lipid metabolism, hypertension, nutritional and endocrine-metabolic conditions, anxiety, depression and mood disorders. During the first four years of the follow-up, 4,895 individuals died and 10,003 were lost to follow-up; the cluster analysis in 2015 included a total of 279,025 individuals. During the last four years of the follow-up, 5,680 individuals died and 2,072 were lost to follow-up; a total of 271,273 individuals were included in the cluster analysis in 2019. During the whole eight-year follow-up, 10,575 (3.6%) individuals died, and 12,075 (4.11%) were lost to follow-up.

**Table 1:** Demographic and clinical characteristics of all individuals 18–65 years old in the EpiChron Cohort (Aragón, Spain) who had multimorbidity in 2011.^a^

	Total	Women	Men
**Population, *n* (%)**			
2011	293,923 (100)	170,040 (57.85)	123,883 (42.15)
2015	279,025	162,260 (58.15)	116,765 (41.85)
2019	271,273	157,690 (58.13)	113,583 (41.87)
**Mean age (SD)**			
2011	47.77 (12.15)	47.06 (12.42)	48.73 (11.70)
2015	51.83 (12.09)	51.22 (12.36)	52.68 (11.67)
2019	55.98 (12.01)	55.39 (12.27)	56.79 (11.60)
**Chronic diseases, *n* (SD)**			
2011	3.24 (1.55)	3.33 (1.61)	3.12 (1.43)
2015	3.95 (1.96)	4.08 (2.04)	3.77 (1.82)
2019	4.69 (2.29)	4.87 (2.37)	4.45 (2.15)
**Drugs,**^b^ ***n* (SD)**			
2012–2015	11.35 (7.81)	12.41 (8.04)	9.91 (7.22)
2016–2019	11.31 (7.62)	12.24 (7.79)	10.02 (7.16)
**Acute diseases,**^c^ ***n* (SD)**			
2012–2015	4.84 (3.60)	5.45 (3.76)	4.00 (3.14)
2016–2019	4.98 (3.64)	5.59 (3.83)	4.13 (3.19)

a Multimorbidity was defined as the co-existence of ≥2 chronic conditions in the same individual. The table shows the mean age, and the mean number of chronic diseases in each cross-section (2011, 2015, and 2019), as well as the mean number of different drugs and acute diseases during the four-year follow-up periods (2012–2015 and 2016–2019). All data are accompanied with their corresponding standard deviation (SD). Clinical information is based on electronic health records from both primary and specialised care.

b The mean number of the different drugs dispensed per individual during the follow-up, using the World Health Organization’s Anatomical Therapeutic Chemical (ATC) classification system at the fourth ATC level (i.e., chemical subgroup). Repeated dispensations of drugs that were classified in the same fourth ATC level during a follow-up period were counted once.

c The mean number of different acute diseases per individual during the follow-up. Acute conditions that were diagnosed multiple times (two or more times) in a follow-up period were counted once.

By employing cluster analysis, we identified three clusters of individuals with different baseline clinical and epidemiological characteristics (Table 2). A full description of the clinical component of the multimorbidity patterns is available in Table A1. Disorders of lipid metabolism and arterial hypertension were the conditions that played a key role in the clinical differentiation between the clinical profiles of the multimorbidity patterns (Figure A2). The *dyslipidaemia & endocrine-metabolic* pattern, with a mean number of 3.88 ± 1.79 (standard deviation, SD) chronic diseases per individual, included all patients with a diagnosis of dyslipidaemia. Other common diseases in this pattern were nutritional and endocrine-metabolic disorders. Obesity was more common in the *hypertension & obesity* pattern, with a mean number of 2.69 ± 1.05 chronic diseases. In the absence of dyslipidaemia, individuals with a diagnosis of arterial hypertension or obesity were more likely to belong to this pattern. All other individuals were included in an *unspecific* pattern with, on average, 3.08 ± 1.38 chronic diseases per individual. Menstrual disorders were amongst the most common comorbidities in the *unspecific* pattern, explaining partly that most of the individuals of this pattern (73.17%) were females. Other common comorbidities in this pattern were headache and migraine, anxiety, upper respiratory disease, and thyroid disorders. The patients of the *unspecific* pattern were the youngest, and they had a lower prevalence of nutritional and endocrine-metabolic disorders (except of thyroid disorders) in comparison with the individuals of other patterns.

**Table 2:** Demographic and clinical characteristics of the multimorbidity patterns in adults 18–65 years old with multimorbidity in the EpiChron Cohort (Aragón, Spain) in 2011.^a^

	Dyslipidaemia & endocrine-metabolic	Hypertension & obesity	Unspecific
Size, *n* (%)	110,594 (37.63)	73,236 (24.92)	110,093 (37.46)
Women, *n* (% distribution)	51,137 (30.07)	38,344 (22.55)	80,559 (47.38)
Mean age (SD)	52.01 (9.92)	52.03 (10.47)	40.66 (11.89)
Chronic diseases, *n* (SD)	3.88 (1.79)	3.08 (1.38)	2.69 (1.05)
Chronic diseases in 2015, *n* (SD)	4.57 (2.12)	3.99 (1.91)	3.32 (1.60)
Drugs during 2012–2015,^b^ *n* (SD)	11.98 (8.08)	12.15 (8.10)	10.19 (7.16)
Acute diseases during 2012–2015,^c^ *n* (SD)	4.76 (3.50)	4.82 (3.59)	4.94 (3.68)
Four-year mortality,^d^ *n* (%)	1903 (1.72)	1953 (2.67)	1,039 (0.94)
Eight-year mortality,^d^ *n* (%)	4,415 (3.99)	4,056 (5.54)	2,104 (1.91)

a Multimorbidity patterns were identified through *k*-means non-hierarchical clustering in 2011. Multimorbidity was defined as the co-existence of ≥2 chronic conditions in the same individual. Data are accompanied with their corresponding standard deviation (SD). Clinical information is based on electronic health records from both primary and specialised care.

b The mean number of the different drugs dispensed per individual, using the World Health Organization’s Anatomical Therapeutic Chemical (ATC) classification system at the fourth ATC level (i.e., chemical subgroup). Repeated dispensations of drugs that were classified in the same fourth ATC level during a follow-up period were counted once.

c The mean number of acute diseases per individual. Acute conditions that were diagnosed multiple times (two or more times) were counted once.

d Four-year mortality was assessed in 2015. Eight-year mortality was assessed in 2019.

### 3.2 The evolution of the multimorbidity patterns

**Figure 1:**
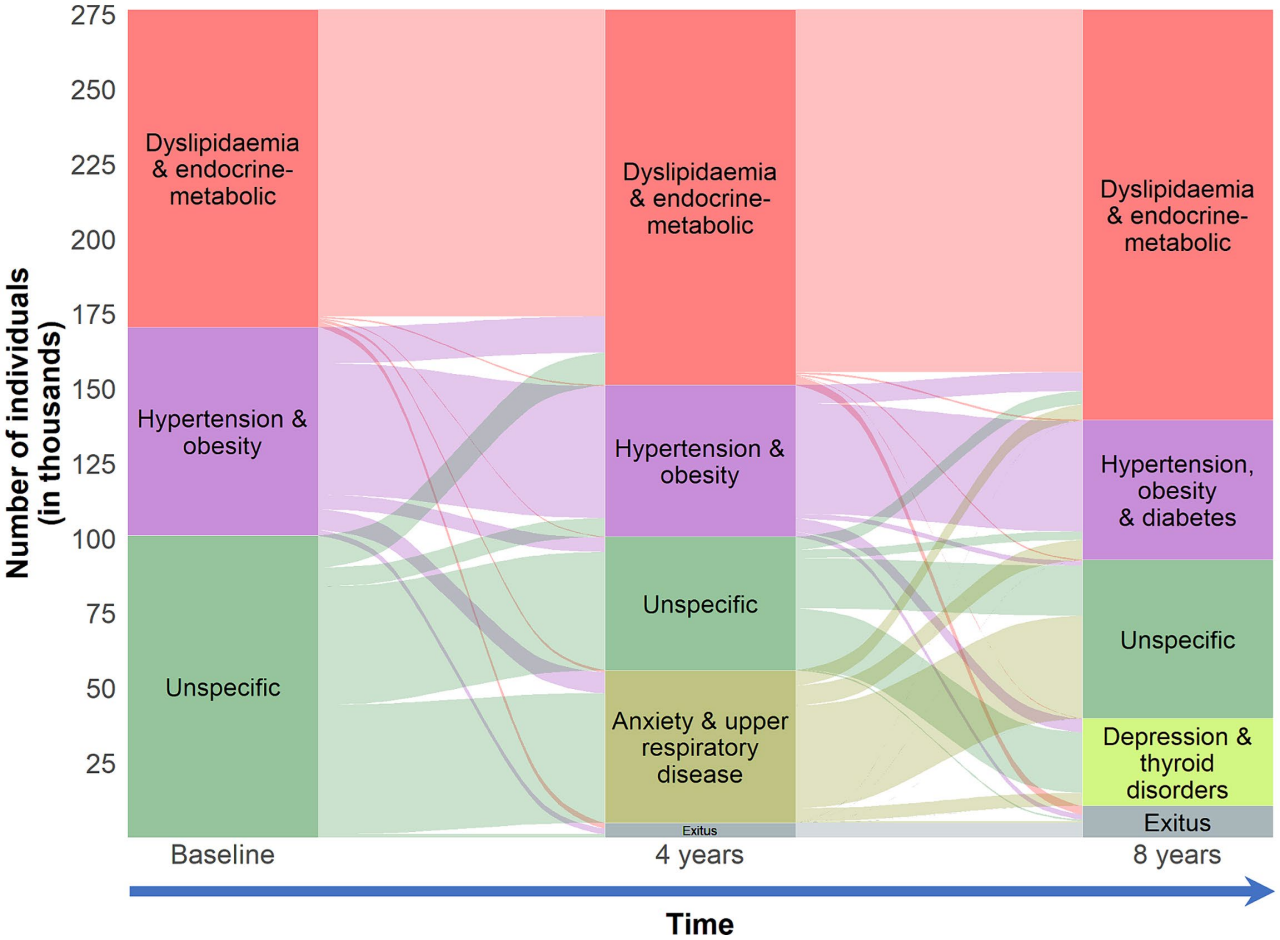
Clinical trajectories of individuals 18–65 years old in the EpiChron Cohort (Aragón, Spain) who had multimorbidity at baseline (2011). Multimorbidity was defined as the co-existence of ≥2 chronic conditions in the same individual. Clinical information is based on electronic health records from both primary and specialised care. The alluvial plot depicts the evolution of the multimorbidity patterns considering both four-year follow-up periods. The boxes represent the multimorbidity patterns identified through *k*-means non-hierarchical clustering at baseline, and then at the fourth (2015) and eighth (2019) year of follow-up. The stripes represent multimorbidity trajectories across the multimorbidity patterns during the follow-up (the number of individuals who evolved from one multimorbidity pattern to another). The size of the features (boxes and stripes) is proportional to the number of individuals in the corresponding pattern/trajectory.

**Figure 2:**
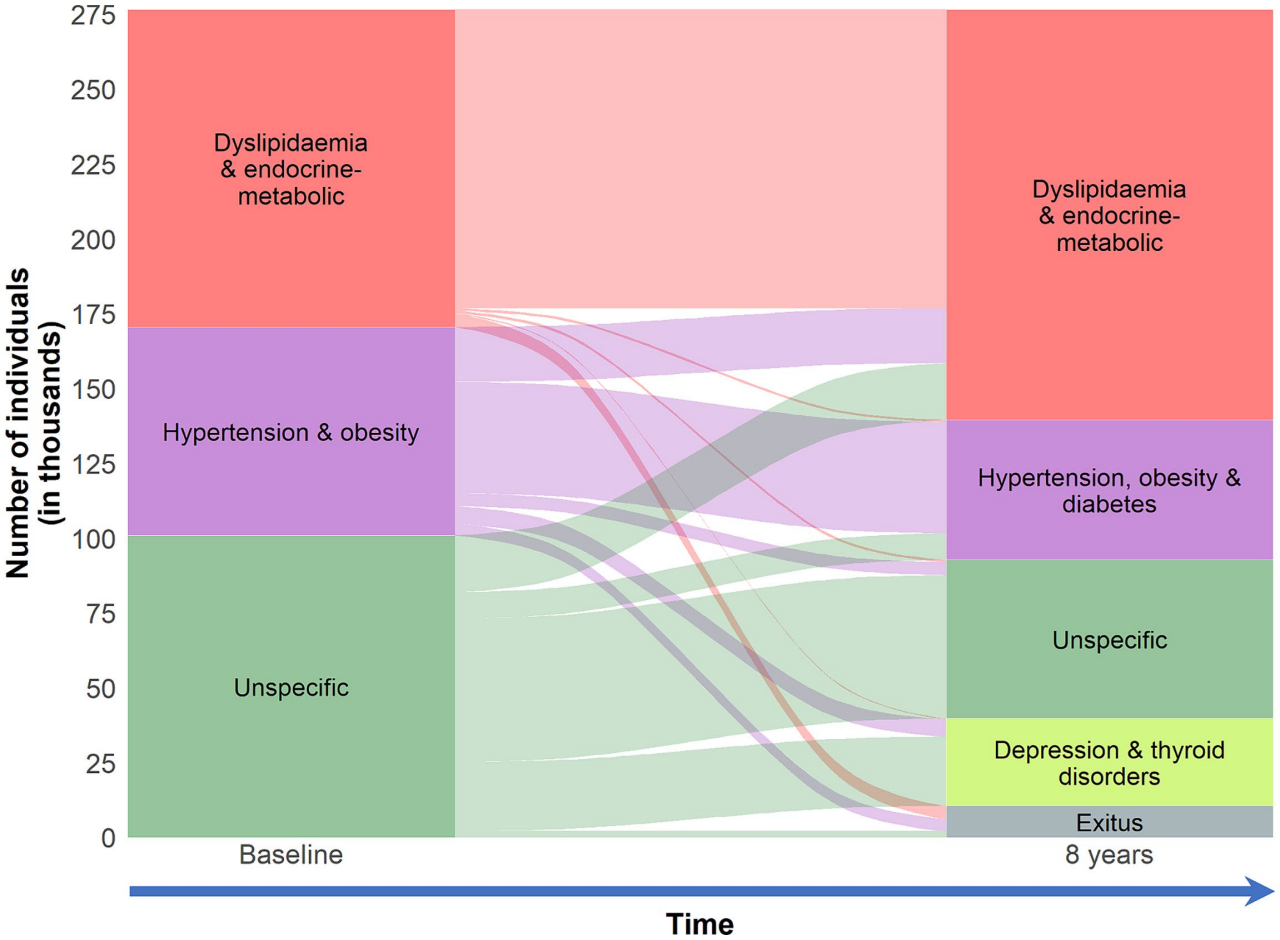
Eight-year clinical trajectories of individuals 18–65 years old in the EpiChron Cohort (Aragón, Spain) who had multimorbidity at baseline (2011). Multimorbidity was defined as the co-existence of ≥2 chronic conditions in the same individual. Clinical information is based on electronic health records from both primary and specialised care. The alluvial plot depicts the evolution of the multimorbidity patterns at eight years from baseline. The boxes represent the multimorbidity patterns identified through *k*-means non-hierarchical clustering in 2011 and 2019. The stripes represent multimorbidity trajectories across the multimorbidity patterns during the follow-up (the number of individuals who evolved from one multimorbidity pattern to another). The size of the features (boxes and stripes) is proportional to the number of individuals in the corresponding pattern/trajectory.

The mean number of chronic conditions increased with time during the follow-up (Table 1). With cluster analysis, we identified four multimorbidity patterns in 2015 and four patterns in 2019 (Table 3). The full description of the clinical component of the multimorbidity patterns is available in Table A1. In this longitudinal study, we were able to identify different four- and eight- year multimorbidity trajectories, each with its particular demographic and clinical characteristics (Figures 1, 2 and Table A2).

**Table 3:** Demographic and clinical characteristics of the multimorbidity patterns at the fourth (2015) and the eighth (2019) year of follow-up in adults 18–65 years old in the EpiChron Cohort (Aragón, Spain) who had multimorbidity at baseline (2011).^a^

Multimorbidity patterns in 2015	Dyslipidaemia & endocrine-metabolic	Hypertension & obesity	Unspecific	Anxiety & upper respiratory disease
Size, *n* (%)	127,665 (45.75)	51,690 (18.53)	46,493 (16.66)	53,177 (19.06)
Women, *n* (% distribution)	63,271 (38.99)	25,312 (15.60)	38,471 (23.71)	35,206 (21.70)
Mean age (SD)	56.02 (9.79)	56.28 (10.25)	44.64 (11.98)	43.74 (11.75)
Chronic diseases, *n* (SD)	4.70 (2.08)	3.58 (1.74)	3.07 (1.36)	3.26 (1.57)
Chronic diseases in 2019, *n* (SD)	5.44 (2.39)	4.51 (2.17)	3.85 (1.84)	3.94 (1.98)
Drugs during 2012–2015,^b^ n (SD)	12.26 (7.77)	11.97 (7.86)	9.85 (7.02)	9.68 (7.02)
Acute diseases during 2012–2015,^c^ *n* (SD)	5.06 (3.58)	4.94 (3.62)	4.98 (3.75)	4.83 (3.74)
Four-year mortality,^d^ *n* (SD)	2,960 (2.32)	1,645 (3.18)	491 (1.06)	554 (1.04)

Multimorbidity patterns in 2019	Dyslipidaemia & endocrine-metabolic	Hypertension, obesity & diabetes	Unspecific	Depression & thyroid disorders
Size, *n* (%)	139,089 (51.27)	48, 368 (17.83)	53,789 (19.83)	30,027 (11.07)
Women, *n* (% distribution)	71,697 (45.47)	24,099 (15.28)	38,729 (24.56)	23,165 (14.69)
Mean age (SD)	59.71 (9.84)	60.13 (10.13)	44.50 (10.95)	52.58 (11.71)
Chronic diseases, *n* (SD)	5.48 (2.35)	4.43 (2.09)	3.46 (1.64)	3.71 (1.81)
Drugs during 2016–2019,^b^ *n* (SD)	12.26 (7.77)	9.85 (7.02)	9.68 (7.02)	11.97 (7.86)
Acute diseases during 2016–2019,^c^ *n* (SD)	6.55 (4.85)	6.37 (4.94)	3.85 (3.75)	4.93 (4.35)

a Multimorbidity patterns were identified through *k*-means non-hierarchical clustering in 2015 and 2019, respectively. Multimorbidity was defined as the co-existence of ≥2 chronic conditions in the same individual. Data are accompanied with their corresponding standard deviation (SD). Clinical information is based on electronic health records from both primary and specialised care.

b The mean number of the different drugs dispensed per individual, using the World Health Organization’s Anatomical Therapeutic Chemical (ATC) classification system at the fourth ATC level (i.e., chemical subgroup). Repeated dispensations of drugs that were classified in the same fourth ATC level during a follow-up period were counted once.

c The mean number of acute diseases per individual. Acute conditions that were diagnosed multiple times (two or more times) were counted once.

d Assessed in 2019.

Over the eight years of follow-up, we were able to identify a *dyslipidaemia & endocrine-metabolic* pattern. Approximately seven in ten individuals in this pattern in 2019 came from a similar pattern back in 2011 (98.1% of those individuals). The mean number of chronic conditions in these patterns was 4.70 ± 2.08 and 5.48 ± 2.35 in 2015 and 2019, respectively. Amongst the most common conditions were nutritional and endocrine-metabolic disorders and hypertension. The prevalence of hypertension increased in these patterns from 34 to 48% during the eight-year follow-up.

We identified an *hypertension & obesity* pattern in 2015 and an *hypertension, obesity & diabetes* pattern in 2019, with an average of 3.58 ± 1.74 and 4.43 ± 2.09 chronic diseases, respectively. None of these individuals had a diagnosis of dyslipidaemia during the whole follow-up. However, the prevalence of hypertension, obesity, and diabetes in the *dyslipidaemia & endocrine-metabolic* pattern increased with time. Approximately, four out of every five individuals of the *hypertension, obesity & diabetes* pattern in 2019 came from *hypertension & obesity* pattern back in 2011 (57.19% of those individuals).

Most of the individuals who belonged to the *unspecific* pattern at baseline were split into two main patterns after a four-year follow-up. The main chronic conditions that were key diagnoses in this “unspecific-profile pattern separation” were anxiety and upper respiratory disease, giving birth to *anxiety and upper respiratory disease* pattern with an average of 3.26 ± 1.57 chronic conditions per patient. Another common comorbidity in this pattern was asthma. Approximately, 85% of the individuals of this pattern came from the *unspecific* pattern back in 2011. Approximately, 44% of the 2011 *unspecific* pattern evolved to *anxiety and upper respiratory disease* pattern; the rest was mostly remained to an *unspecific* pattern in 2015, with an average of 3.07 ± 1.36 chronic diseases per patient. Amongst the most common chronic conditions were menstrual disorders, headache, migraine, thyroid disorders, depression, and mood disorders. During the second four-year follow-up period, we saw that two other clinical diagnoses played a key role in the “unspecific-profile pattern separation” while anxiety and upper respiratory disease were associated again with the new “classic” *unspecific* pattern in 2019, together with other common comorbidities such as menstrual disorders, headache, migraine, and asthma; individuals in this pattern had, on average, 3.46 ± 1.64 chronic diseases. Depression, mood disorders, and thyroid disorders were the main conditions of *depression and thyroid disorders* pattern, with a mean of 3.71 ± 1.81 chronic diseases per individual.

### 3.3 Acute diseases during the follow-up

The presence of acute diseases during the previous four-year follow-up was studied for all 2015 and 2019 multimorbidity patterns (Table A3). The number of acute diseases during the follow-up was higher in women than in men, with approximately 5.5 and 4.0 acute diseases, respectively, during a four-year follow-up (Table 1). Upper respiratory infections were the most common acute diseases, found in approximately half of the individuals during the four-year follow-up periods. Other common acute diseases were non-traumatic joint disorders, back problems, urinary tract and intestinal tract infections, and acute diseases of teeth and jaw. Age- and sex-adjusted multinomial regression analysis was carried out to find possible associations between acute diseases and multimorbidity patterns (Table A4). In the second half of the follow-up, we saw that nutritional deficiencies, upper respiratory infections, and back problems were associated with the *dyslipidaemia & endocrine-metabolic* pattern.

### 3.4 Drug utilisation during the follow-up

In both four-year follow-up periods, the number of different drugs was higher in women than in men (Table 1). The multimorbidity patterns in 2015 and 2019 with the highest mean number of dispensed drugs were the *dyslipidaemia & endocrine-metabolic* and the “hypertensive” patterns (*hypertension & obesity* in 2015, and *hypertension, obesity and diabetes* in 2019). Propionic acid derivatives (for example, ibuprofen, naproxen, and dexketoprofen), benzodiazepine derivatives, proton pump inhibitors, and anilides (for example, paracetamol) were amongst the most common drugs in all multimorbidity patterns (Table A4). HMG CoA reductase inhibitors, commonly known as statins, were dispensed in half of the individuals of the *dyslipidaemia & endocrine-metabolic* patterns (in both four-year follow-up periods) while in the “hypertensive” patterns the prevalence was between 13 and 16% in the first and the second four-year follow-up, respectively. Besides statins, other commonly dispensed drugs for the cardiovascular system in these multimorbidity patterns were agents acting on the renin-angiotensin system, mostly angiotensin-converting-enzyme (ACE) inhibitors and angiotensin II receptor blockers (ARBs), the latest, plain or in combination with diuretics. Dispensation of these drugs were higher during the second four-year follow-up in both multimorbidity patterns. The most dispensed antidiabetic drugs were biguanides; approximately 9% in the “hypertensive” patterns, and 8% in the *dyslipidaemia & endocrine-metabolic* patterns, followed by combinations of oral antidiabetics, and then, insulins and analogues.

### 3.5 Mortality

The *hypertension and obesity* pattern had the highest four- and eight- year (age- and sex-adjusted) mortality risk of the three baseline multimorbidity patterns (Table 4). Considering the pattern with the lowest mortality as reference, the odds of mortality to occur amongst individuals in the *hypertension and obesity* pattern were approximately 16% higher, adjusted for age and sex.

**Table 4:** Age- and sex-adjusted all-cause mortality risk for all multimorbidity patterns identified in 2011 and 2015 in adults 18–65 years old in the EpiChron Cohort (Aragón, Spain) who had multimorbidity at baseline (2011).^a^

	Mortality	
	Events, *n* (%)	OR (95% CI)
**8 years from baseline (2012–2019)**		
Dyslipidaemia & endocrine-metabolic	4,415 (3.99)	0.81 (0.76–0.85)
Hypertension & obesity	4,056 (5.54)	1.16 (1.10–1.23)
Unspecific	2,104 (1.91)	ref.
**4 years from baseline (2012–2015)**		
Dyslipidaemia & endocrine-metabolic	1903 (1.72)	0.70 (0.64–0.76)
Hypertension & obesity	1953 (2.67)	1.11 (1.02–1.21)
Unspecific	1,039 (0.94)	ref.
**4 years from 2015 (2016–2019)**		
Dyslipidaemia & endocrine-metabolic	2,960 (2.32)	0.88 (0.80–0.96)
Hypertension & obesity	1,645 (3.18)	1.15 (1.04–1.28)
Unspecific	491 (1.06)	1.10 (0.97–1.24)
Anxiety & upper respiratory disease	554 (1.04)	ref.

a Multimorbidity patterns were identified by *k*-means non-hierarchical clustering. Multimorbidity was defined as the co-existence of ≥2 chronic conditions in the same individual. Clinical information is based on electronic health records from both primary and specialised care. Information regarding mortality (assessed in 2015 and 2019) is given in number of events (*n*), % prevalence, and odds ratio (OR) with the corresponding 95% confidence interval (95% CI), considering the pattern with the lowest mortality as reference.

## 4 Discussion

### 4.1 Discussion of the main results

This study confirmed that multimorbidity is adults’ most common clinical presentation of chronicity. Cluster analysis allowed us to identify three multimorbidity patterns at baseline and study their clinical evolution over the following eight years. Dyslipidaemia and hypertension were important in determining the clinical particularities of these patterns. We identified different multimorbidity trajectories, with some consistency over the eight years of follow-up. Inflammatory chronic conditions were highly prevalent in all multimorbidity patterns and trajectories, especially the cardiometabolic ones. We saw differences in drug utilisation between the multimorbidity patterns and identified signals of potential drug interactions that could result in severe outcomes. We also analysed the presence of acute diseases, finding some possible associations with specific patterns.

In the older population, it is well-known that multimorbidity is the rule rather than the exception (7–12, 24, 53). This study showed that multimorbidity is a challenging situation for all the adult population, being the most common clinical presentation of chronicity. Our data revealed that most chronic patients 18–65 years old have multiple chronic conditions. Even though the prevalence of multimorbidity increases with age, most people with multiple chronic conditions are adults 18–65 years old. Our findings emphasise the importance of the recommendations of the Academy of Medical Sciences (United Kingdom) to advance research in the field (22, 23); for example, to study in-depth trends and patterns of multimorbidity, and their impact on health, to offer optimal clinical management and to adequately address the needs of the population at risk.

By applying cluster analysis, we saw that dyslipidaemia and arterial hypertension are crucial in clustering the population based on an individual’s clinical profile. Two out of every three chronic patients with multiple chronic conditions had a cardiovascular clinical profile during the follow-up. Almost all (99%) individuals from the *dyslipidaemia & endocrine-metabolic* and most (approximately 85%) of those from the *hypertension & obesity* pattern remained in a cardiovascular pattern (either *dyslipidaemia & endocrine-metabolic* or an “hypertensive” pattern) in the next eight years from baseline. The high prevalence of these patterns is a concerning finding, considering that key conditions are well-known important—and preventable—cardiovascular risk factors (65–70). Amongst the most common diseases of these patterns were diabetes and obesity. The systematic associations between these conditions with other chronic cardiovascular diseases are consistently reported in the medical literature. Especially in the *dyslipidaemia & endocrine-metabolic* pattern, patients face the risk of metabolic syndrome and its critical clinical consequences it may further add, especially in patients with a high morbidity burden (71). We saw, for example, that in the *dyslipidaemia & endocrine-metabolic* pattern—where each patient has approximately five chronic diseases—the prevalence of arterial hypertension increased considerably, being present in half of the pattern’s population in 2019. In addition, an important finding was that various chronic conditions particularly prevalent in the cardiometabolic patterns involve chronic inflammation; such diseases include diabetes, obesity, heart disease, atherosclerosis, arthritis, cancer, and chronic obstructive pulmonary disease. In a previous study, network analysis showed that cardio-metabolic conditions, respiratory diseases, and chronic kidney failure were highly connected in all multimorbidity networks in patients with heart failure and/or chronic obstructive pulmonary disease (72).

In this study, we presented data regarding the diagnoses of acute diseases during the follow-up. Each patient had, on average, five different acute diseases in both four-year follow-up periods. We saw a high prevalence of infections, mainly upper-respiratory (approximately half of the population), urinary, intestinal tract, and eye infections; mycoses were also common findings. The second most common acute disease was related to back problems (spondylosis and intervertebral disc disorders), observed in one out of every four patients. We saw that certain acute diseases were associated with specific trajectories, when adjusting for age and sex. For example, in the second half of the follow-up, nutritional deficiencies, upper respiratory infections, and back problems were associated with the *dyslipidaemia & endocrine-metabolic* pattern. Further research is needed to confirm if these findings are beyond chance and, if so, to study the nature of these associations and their impact on health.

Approximately one out of every five patients had at least one acute disorder related to teeth and jaw during the first four years of the follow-up. In Primary Care, mouth and tooth disorders diagnoses are mostly acute medical conditions, such as abscesses, and are well-documented in EHR. However, chronic oral diseases, such as periodontitis, usually are not a reason for consultation with the general practitioner and, consequently, are not well-documented in EHR. Many stomatognathic diseases are linked to chronic inflammation and associated with a higher risk of additional comorbidity, mainly cardiovascular and cerebrovascular conditions (73–76). Sometimes inflammatory oral diseases may be perceived by the patient as “innocent” or “insignificant,” avoiding consultation with a healthcare professional. Untreated aetiology can turn acute inflammation into an insidious form of chronic inflammation (77). It is, therefore, crucial to promote oral hygiene, establish preventive programmes for oral health in the community, and strengthen oral medicine in Primary Healthcare services.

Amongst the most common medications in all multimorbidity trajectories were proton pump inhibitors, propionic acid derivatives (for example, ibuprofen, naproxen, and dexketoprofen), anilides (for example, paracetamol), and benzodiazepine derivatives. These medications are highly prevalent in patients with chronic polypharmacy (78). We saw differences in drug utilisation between the patterns. Individuals with a cardiometabolic profile use a wider variety of medications, compared with the *unspecific* patterns.

In the *anxiety and upper respiratory disease* pattern, benzodiazepine derivatives and combinations of adrenergics with corticosteroids (inhalants) were amongst the most common medications. A common adverse drug reaction of these agents is gastro-oesophageal reflux disease (79, 80), which may result in additional risks; for example, respiratory infections. It is unsurprising to find proton-pump inhibitors among this pattern’s most common medications. It has also been reported that benzodiazepine derivatives may induce depressive symptoms (81), which could partly explain some of the prescriptions of antidepressants in this same pattern. Furthermore, selective serotonin reuptake inhibitors may increase the risk of gastrointestinal disorders. Especially in combination with nonsteroidal anti-inflammatory drugs, there is a considerably higher risk of gastrointestinal bleeding (82–84).

In the *dyslipidaemia & endocrine-metabolic* patterns, statins are the most common medications. Well-known adverse reaction of statins are myalgias, which may be treated with nonsteroidal anti-inflammatory agents, partly explaining that propionic acid derivatives were amongst the most common pharmacological groups of the pattern. We also find dispensations of antibiotics that could be prescribed to eradicate *Helicobacter pylori* in combination with proton pump inhibitors. We see an exceptionally high prevalence of proton pump inhibitors in cardiometabolic patterns. It has been reported that this group of gastroprotectors can reduce the absorption of vitamin B12, potentially resulting in vitamin B12 deficiency (85, 86). A possible increase in homocysteine could lead to a higher risk of cardiovascular complications (87, 88), such as thrombosis.

An unexpected finding of particular concern in the hypertensive patterns was the high prevalence of propionic acid derivatives, even higher than the ones for angiotensin-converting enzyme inhibitors, and angiotensin II receptor blockers and diuretics. Nonsteroidal anti-inflammatory agents may block the effects of antihypertensives and result in poor blood pressure control (89, 90). This is of extreme importance, considering that the hypertensive patterns, with a high prevalence of arterial hypertension (80% in 2019), obesity, and diabetes mellitus, are the patterns that were associated with a higher mortality risk. In addition, considering the high prevalence of propionic acid derivatives, there is a high risk of interactions between angiotensin-converting enzyme inhibitors or angiotensin II receptor blockers, diuretics, and nonsteroidal anti-inflammatory agents—commonly known as triple whammy—which increases the risk of kidney failure (91).

So, although many studies and interventions have been conducted to characterise and tackle multimorbidity, the underlying mechanisms in its development and clinical evolution still need to be discovered while its prevalence is continuously on the rise. Additional morbidity and complex interactions between diseases (acute and chronic) and drugs are associated with negative outcomes and a higher risk of fragmented care. Following our results and emerging evidence, there are several ways to reduce some risks; for example, by promoting healthy and active ageing (see examples of general tips in Table 5) (77, 92–103), preventing hypertension and other cardiovascular risk factors, preventing common risk factors for systemic chronic inflammation, promoting interdisciplinary teamwork in everyday clinical praxis, regularly reviewing patients’ medication lists, informing the patient adequately regarding medication use and potentially severe interactions with drugs that can commonly be taken over the counter (self-medications), and promoting oral-health with specific preventive programmes. It is essential to work towards a comprehensive and interdisciplinary approach, where various healthcare professionals can interact, collaborate, and coordinate person-centred care models through integrated health and social services.

**Table 5:** Some general tips to prevent the onset or progression of multimorbidity and promote healthy and active ageing.

Tips to prevent the onset or progression of multimorbidity and promote healthy and active ageing
Maintain a healthy and balanced diet (for example, a Mediterranean-style diet)
Avoid sugar, salt, and saturated fat
Prioritise nutrient-rich food and avoid ultra-processed food and unhealthy additives
Maintain a good hydration
Do not exceed your daily calorie allowance
Maintain your body weight between the recommended limits; consult your doctor
Exercise regularly
Get enough quality sleep
Keep the brain active (for example, by learning something new)
Connect with nature
Improve your social life, build good relationships, and connect with friends
Experience a sense of meaning and purpose (for example, by volunteering)
Reduce chronic stress
Visit regularly your doctor and your dentist
Talk with your doctor about any symptoms and changes you have noticed, physical and mental
Know your family’s medical history
Regularly check your blood pressure
Practice good oral hygiene
Have a good understanding of your health issues and treatment plans
Collaborate with your doctor and get involved in setting goals for your health
Have a good understanding of your medication
Let your doctor know about medications you have used over the counter
Be aware of all preventive programmes that are suitable for your age; consult your doctor
Practice safe sex
Do not smoke
Avoid alcohol
Speak up if you feel down

### 4.2 Comparison with previous studies

Previous studies in multimorbidity followed a cross-sectional approach to identify common clinical patterns (26, 30). Most of these studies focus on older adults, mainly because multimorbidity prevalence is higher in this age group (11, 12, 38). Despite some discrepancies, partly explained by differences in the methodological approaches, most studies report a very consistent cardiometabolic pattern with a high morbidity burden. In advanced age (80 years and older), the prevalence of some metabolic diseases decreases, for example, for diabetes and dyslipidaemia (104, 105). This could indicate higher mortality rates for individuals with these diagnoses in younger age groups. It seems that the age of onset of the diseases may be a determining factor for the clinical evolution of the patients (60). It has been hypothesised that the healthier a person has been, the healthier they will age (61). In our study, we saw that two out of every three adults 18–65 years old with multimorbidity had a cardiometabolic profile, and thus, they had at least one inflammatory chronic condition (for example, dyslipidaemia, diabetes, obesity, arterial hypertension). The mean age of the individuals of the cardiometabolic patterns was 52 years. This means that most of the adult population with multiple chronic conditions may live, on average, 13 years with an active inflammatory disease—and high morbidity burden—before age 65.

Longitudinal studies in multimorbidity have primarily focused on older adults. In a Swedish study, the authors applied cluster analysis in a longitudinal cohort of community-dwelling adults 60 years and older and identified six multimorbidity patterns (46). They found that half of the population was in an unspecific pattern, while a pattern of heart diseases included 9.3% at baseline. The other baseline patterns were respiratory and musculoskeletal disease, cognitive and sensory impairment, eye diseases and cancer, and psychiatric and respiratory diseases pattern. Mortality at six years from baseline was more likely to occur in the cognitive and sensory impairment pattern, followed by the heart disease pattern.

Another longitudinal study applied hidden Markov models in older adults 65–99 years old in Spain, based on information from EHR from Primary Care (47). The authors identified 10 multimorbidity patterns at baseline. The most common pattern was the non-specific (42%), and it was named so because it included non-overrepresented diseases such as prostate conditions, arterial hypertension, neoplasms, and dyslipidaemia. During the five years of follow-up, the authors reported higher mortality rates in patterns with cardiovascular conditions; these patterns were cardio-circulatory and renal pattern, nervous, digestive and circulatory pattern, and cardio-circulatory, mental, respiratory and genitourinary pattern.

A large-scale epidemiologic study in adults 45 years and older receiving care at community health centres in the USA between 2012 and 2019 (106), based on 22 groups of chronic diseases, reported that 53.2% of the population with multiple chronic conditions were included at the cardiometabolic multimorbidity pattern. The authors grouped the patients into mutually exclusive patterns according to their coexisting diseases. The cardiometabolic multimorbidity pattern included all patients who had ≥2 cardiovascular or metabolic diseases (for example, cardiac arrhythmia, congestive heart failure, diabetes, hypertension), or ≥1 cardiometabolic disease with ≥1 other somatic disease (for example, arthritis, asthma, obstructive pulmonary disease). The second most prevalent multimorbidity pattern was the mental-somatic multimorbidity which included the 41.5% of the population with multiple chronic diseases. Other patterns were the mental multimorbidity, and the other somatic multimorbidity pattern. From the population with no multimorbidity (zero or one chronic diseases), 19% were shifted to cardiometabolic multimorbidity during the follow-up, while 13.5% to mental-somatic multimorbidity.

Another study in individuals 40 years and older, based on information from electronic health records from Primary Care in Belgium between 1991–2015, applied Markov chains to study multimorbidity trajectories (107). The prevalence of multimorbidity was 67% and amongst the most common conditions were hypertension, dyslipidaemia, depression, and diabetes. The authors reported that these highly prevalent conditions were very likely to occur after the diagnosis of most conditions. Another common finding was the co-ocurrence of diseases from the same disease group; a patient with a certain disease would be at higher risk of developing another disease of the same group. Such groups were, for example, cardiovascular, musculoskeletal, endocrine, and metabolic. The study reported important associations between diseases, as was the case between depressive disorder and irritable bowel disease. The authors reported that hypertension and diabetes were strongly associated with many chronic diseases.

Further research is needed, especially large-scale epidemiologic studies in different populations and clinical settings, to identify and clinically characterise the development and evolution of multimorbidity trajectories in the young and middle-aged population. A better understanding could help us design and implement effective strategies towards healthier ageing.

### 4.3 Limitations

This large-scale epidemiologic cohort study includes all individuals 18–65 years old in our region who have multiple chronic conditions and are users of the Spanish National Health System. Information regarding diagnoses was obtained from EHR; this implies that a physician diagnosed all conditions and were not self-reported by the patients. Data in the EpiChron Cohort continuously undergo quality control check-ups to ensure accuracy and reliability for use in research. All diagnoses for acute and chronic conditions from primary and specialised care—and emergency room—were included in this study to enhance validity. However, some conditions that usually are not a reason for consultation with a physician, may be not well-documented in the EHR, and therefore under-represented; for example, chronic oral diseases (such as periodontitis). Another common limitation in studies using electronic health records, are recording errors. Information regarding drug dispensation was based on pharmaceutical billing records. At the present work, we analysed medication usage to complement the diagnoses information and better characterise the multimorbidity patterns and their trajectories. Further studies are needed to address in-depth the role of medications in the evolution of the multimorbidity patterns. The longitudinal nature of our study allowed us to identify trajectories of multimorbidity during the follow-up. However, we did not consider the time of onset of the chronic conditions at baseline. In our study, we lacked information on various factors that can influence health outcomes, such as biochemical data (lab tests), omics, disease severity, family history, concomitant medication, duration of medication and dosing regimens, initiation and medication adherence, drug use in hospitals and other healthcare institutions, self-medication, non-pharmacological approaches, lifestyle, health-related quality of life, gender, ethnicity, and various demographic and socio-economic characteristics of particular interest. It is necessary to consider all these factors and the above-mentioned limitations in future studies and advance research in this field to understand better the underlying mechanisms—and the role of chronic inflammation—in the development and clinical evolution of multimorbidity and to implement effective interventions for healthy ageing.

## 5 Conclusion

In this large-scale epidemiologic study, we saw how multimorbidity patterns evolve over time in young and middle-aged adults. The patterns found are consistent with the metabolic, mental health, and unspecific patterns usually reported in the literature. Dyslipidaemia and arterial hypertension—very common and well-known preventable cardiovascular risk factors—could determine to which multimorbidity pattern a patient was more likely to belong. Inflammatory chronic conditions were highly prevalent in all multimorbidity patterns and trajectories, especially the cardiometabolic ones, highlighting the need to further study the role of systemic chronic inflammation in the development and evolution of multimorbidity. More studies are encouraged to better characterise the population with an unspecific multimorbidity pattern to help us design and implement effective and comprehensive strategies towards healthier ageing.

## Data availability statement

The data analyzed in this study is subject to the following licenses/restrictions: the datasets analysed during this study are not publicly available because of restrictions imposed by the Aragon Health Sciences Institute (IACS) and asserted by the Clinical Research Ethics Committee of Aragon. Requests to access these datasets should be directed to AG-M, agimenomi.iacs@aragon.es.

## Ethics statement

The studies involving humans were approved by Clinical Research Ethics Committee of Aragón (CEICA; protocol number PI17/0024). The studies were conducted in accordance with the local legislation and institutional requirements. Written informed consent for participation was not required from the participants or the participants’ legal guardians/next of kin in accordance with the national legislation and institutional requirements.

## Author contributions

II-S: Conceptualization, Formal analysis, Investigation, Methodology, Software, Supervision, Visualization, Writing – original draft, Writing – review & editing, Resources. FG-R: Investigation, Writing – review & editing. MA-P-S: Investigation, Writing – review & editing. CL-B: Formal analysis, Software, Writing – review & editing. BP-P: Data curation, Formal analysis, Software, Writing – review & editing. JV-R: Investigation, Writing – review & editing. HC: Investigation, Writing – review & editing. AS-M: Software, Visualization, Writing – review & editing. AP-T: Funding acquisition, Investigation, Resources, Writing – review & editing. AM-J: Investigation, Writing – review & editing. AG-M: Conceptualization, Funding acquisition, Investigation, Methodology, Project administration, Resources, Software, Supervision, Writing – review & editing.
